# Thermodynamic Study of 1,4-Bis(3-methylimidazolium-1-yl)butane Bis(trifluoromethylsulfonyl)imide ([C_4_(MIm)_2_][NTf_2_]_2_) from 6 to 350 K

**DOI:** 10.3390/molecules29174180

**Published:** 2024-09-03

**Authors:** Alexey V. Markin, Andrea Ciccioli, Andrea Lapi, Semen S. Sologubov, Natalia N. Smirnova, Stefano Vecchio Ciprioti

**Affiliations:** 1 Department of Chemistry, National Research Lobachevsky State University of Nizhny Novgorod, Gagarin Avenue 23, 603022 Nizhny Novgorod, Russia; s.slg90@gmail.com (S.S.S.); smirnova@ichem.unn.ru (N.N.S.); 2 Department of Chemistry, Sapienza University of Rome, Piazzale Aldo Moro 5, 00185 Rome, Italy; andrea.ciccioli@uniroma1.it (A.C.); andrea.lapi@uniroma1.it (A.L.); 3 Institute for Biological Systems, Italian National Research Council (ISB-CNR), Secondary Office of Rome-Reaction Mechanisms c/o Department of Chemistry, Sapienza University, Piazzale Aldo Moro 5, 00185 Rome, Italy; 4 Department of Basic and Applied Science for Engineering (S.B.A.I.), Sapienza University of Rome, Via del Castro Laurenziano 7, 00161 Rome, Italy; stefano.vecchio@uniroma1.it

**Keywords:** dicationic ionic liquids, NTf_2_ anion, heat capacity, adiabatic calorimetry, standard thermodynamic functions

## Abstract

The molar heat capacity of 1,4-bis(3-methylimidazolium-1-yl)butane bis(trifluoromethylsulfonyl)imide dicationic ionic compound ([C_4_(MIm)_2_][NTf_2_]_2_) has been studied over the temperature range from 6 to 350 K by adiabatic calorimetry. In the above temperature interval, this compound has been found to form crystal, liquid, and supercooled liquid. For [C_4_(MIm)_2_][NTf_2_]_2_, the temperature of fusion *T*°_fus_ = (337.88 ± 0.01) K has been determined by the fractional melting experiments, the enthalpy of fusion Δ_fus_*H*° = (52.79 ± 0.28) kJ mol^−1^ has been measured using the calorimetric method of continuous energy input, and the entropy of fusion Δ_fus_*S*° = (156.2 ± 1.7) J K^−1^ mol^−1^ has also been evaluated. The standard thermodynamic functions of the studied dicationic ionic compound, namely, the heat capacity *C*_p_°(*T*), the enthalpy [*H*°(*T*) − *H*°(0)], the entropy *S*°(*T*) and the Gibbs free energy [*G*°(*T*) − *H*°(0)] have been calculated on the basis of the experimental data for the temperature range up to 350 K. The results have been discussed and compared with those available in the literature and in the NIST Ionic Liquids Database (ILThermo) for monocationic ionic compounds.

## 1. Introduction

**Figure 1:**
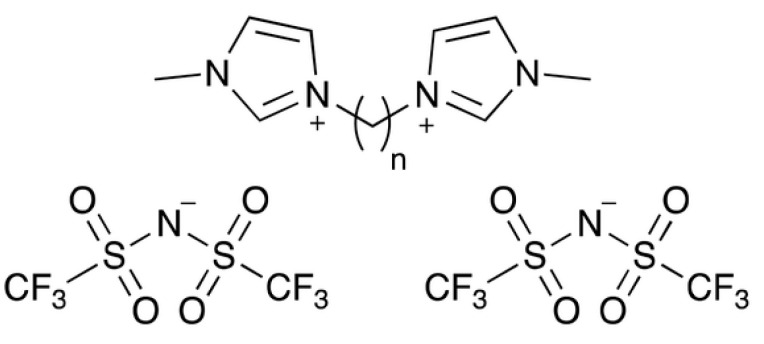
General formula of imidazolium-based DILs with NTf_2_ anions.

The fields of interest in ionic liquids (ILs) include chemical synthesis and catalysis, pharmaceutics and medicine, electrochemistry, biomass conversion, fuel production and processing, liquid crystal development, material science, and many others [1,2,3]. Since the beginnings of the “modern age” of research on ILs, the family of organic salts containing a 1,3-dialkyl imidazolium cation and the bis(trifluoromehtyl)sulfonylimide (NTf_2_) anion has been one of most widely investigated among aprotic ILs [4,5,6,7], although more recently the research involving ILs has been mainly focused on greener compounds. This is in part due to the high thermal stability of these compounds, which is related to the scarce nucleophilicity of the anionic portion [8,9]. Indeed, if the well-known low volatility of ILs is not accompanied by sufficiently high thermal stability, a number of potential applications of this wide class of compounds cannot be exploited. In the search for improved variants of classic alkylimidazolium NTf_2_ ILs with lower volatility and good thermal stability at relatively high temperatures, ILs with a dipositive dication, such as that reported in Figure 1, were more recently considered (commonly known as dicationic ionic liquids, DILs) [10,11]. In these compounds, the doubly charged cation contains two alkylimidazolium rings linked by a (CH_2_)*_n_* chain, with *n* going typically from 1 to 10–12. It was recently shown that DILs have a number of peculiar physical and chemical properties compared to their monocationic counterparts [12]. For example, imidazolium-based DILs exhibit a significant increase in the melting temperature, which can arise from π–π stacking.

Despite this interest, the thermodynamic characterization of DILs is presently very scarce in the literature. To the best of our knowledge, the only studies reported so far were focused on the evaporation of the compound with the *n* = 3 linker and a methyl group on the imidazolium rings [13,14]. In particular, no study has been reported on the heat capacity of any member of this family, and no thermodynamic functions are presently available in both the condensed and vapor phases. Indeed, due to the lack of these data, the shifting of evaporation and sublimation enthalpies from the experimental temperatures to the reference temperature of 298.15 K had to be carried out on the basis of empirical estimates, with a likely loss of accuracy.

In order to begin filling this gap, we present here the first experimental determination of the heat capacity of 1,4-bis(3-methylimidazolium-1-yl)butane bis(trifluoromethylsulfonyl)imide ([C_4_(MIm)_2_][NTf_2_]_2_) in the temperature range from 6 to 350 K by classic adiabatic calorimetry and we derive the thermodynamic functions therefrom. For the sake of comparison, the prototypical monocationic IL, [C_4_MIm][NTf_2_], whose heat capacity values are available in the literature in the entire temperature range [15], was also subjected to the same measurements. Previously, the results of NMR analysis for [C_4_(MIm)_2_][NTf_2_]_2_ were reported in [16].

## 2. Results and Discussion

### 2.1. Heat Capacity Measurements

**Figure 2:**
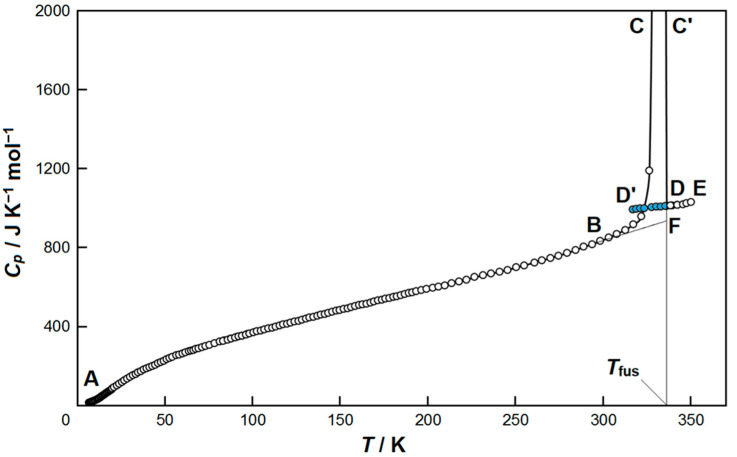
The heat capacity of [C_4_(MIm)_2_][NTf_2_]_2_ in the temperature range 6–350 K: *T*_fus_ is the temperature of fusion; ABF—crystalline state; DE—liquid state; DD’—supercooled liquid state (colored circles); BCC’D—an apparent heat capacity in the interval of fusion.

In order to check the internal consistency of the results of adiabatic measurements, similar to what was reported in a recently published study [17], a set of the heat capacity values was collected for the prototypic 1-butyl-3-methylimidazolium bis(trifluoromethyl)sulfonylimide monocationic ionic liquid [C_4_MIm][NTf_2_] (Table S1, Supplementary Material) under the identical operative conditions used for the tested compound [C_4_(MIm)_2_][NTf_2_]_2_. The *C*_p_ values of three experimental runs for [C_4_MIm][NTf_2_] are compared in Figure S1 (Supplementary Material) with those available in the literature [15]. A very good agreement was found between the experimental and the literature values (Figure S2, Supplementary Material), thus confirming that reliable heat capacity values may also be expected for [C_4_(MIm)_2_][NTf_2_]_2_. The experimental *C*_p_ values of [C_4_(MIm)_2_][NTf_2_]_2_ were determined in the range of 6–350 K, and the plot of the fitted values (*C*_p_ vs. *T*) are shown in Table 1 and Figure 2, respectively. The experimental *C*_p_ values were fitted in all the temperature regions where any transformations are absent according to the following polynomial Equations (1)–(3):

ln*C*_p_ = Σ*A*_i_ · ln(*T*/30)^i^, (1)

*C*_p_ = Σ*A*_i_ · ln(*T*/30)^i^, (2)

*C*_p_ = Σ*A*_i_ · (*T*/30)^i^ (3)

where *A*_i_ is the fitting polynomial coefficient, and *n* is the number of coefficients (starting from 0). The corresponding fitting coefficients are listed in Table S2 (Supplementary Material). The relative deviation of the experimental data from the fitting values related to the tested compound is plotted in Figure S3 (Supplementary Material).

The sample of ionic compound [C_4_(MIm)_2_][NTf_2_]_2_ was cooled down from the room temperature (*T*~298.15 K) to *T*~6 K by liquid helium. At the subsequent heating, the heat capacity of [C_4_(MIm)_2_][NTf_2_]_2_ gradually increases in the temperature range from *T* = (6 to 300) K. Then, the fusion of the studied compound was observed.

### 2.2. Thermodynamic Characteristics of Fusion and the Purity Determination

The enthalpy of fusion Δ_fus_*H*° = (52.79 ± 0.28) kJ mol^−1^ of the dicationic ionic liquid [C_4_(MIm)_2_][NTf_2_]_2_ was determined using the calorimetric method of continuous energy input. The obtained results are given in Table 2.

**Figure 3:**
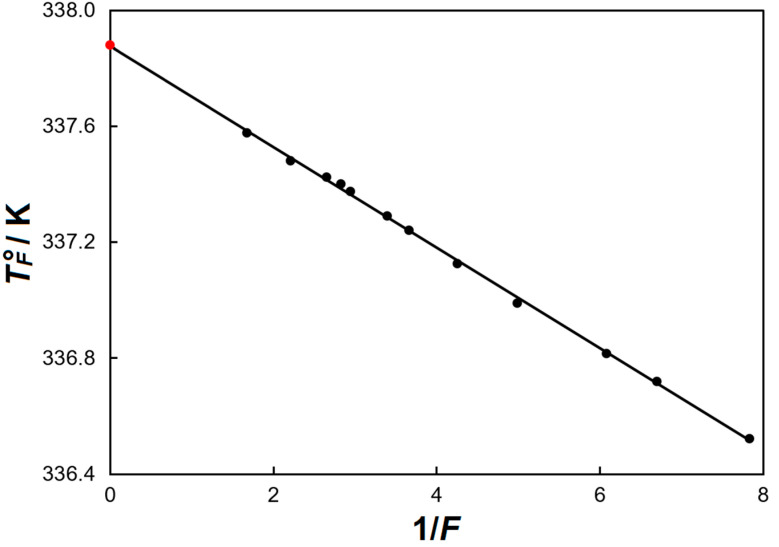
Results of the fractional melting experiment for dicationic ionic liquid [C_4_(MIm)_2_][NTf_2_]_2_.

The mole fraction of impurities in the studied sample (*x*_2_) and the triple point temperature (*T*_tp_) of DIL were determined by the fractional melting experiment (from the calorimetric study of the reciprocal fractions of the sample melted, *F*^−1^, as a function of the equilibrium fusion temperature, *T*°*_F_*) [18]. In other words, at each *T*°*_F_* temperature, the fraction *F* of the area under the calorimetric melting peak is evaluated. The obtained results are listed in Table 3 and are shown in Figure 3.

It can be seen that the corresponding dependence *T*°*_F_* vs. *F*^−1^ is a straight line. It is well described by the Rossini Equation (4):

*T*°*_F_* = *T*°_0_ − *F*^−1^ · (*T*°_0_ − *T*°_1_) (4)

where *T*°_0_ is the triple point temperature of the absolutely pure compound, and *T*°_1_ is the triple point temperature of the completely melted substance (*F* = 1). The deviation of the experimental *T*°*_F_* points from the dependence *T*°*_F_* = *f*(*F*^−1^) does not exceed 0.001%. The obtained values (*T*°_0_ = (337.88 ± 0.01) K and *T*°_1_ = (337.70 ± 0.01) K) are equal to the *T*°_fus_ of pure [C_4_(MIm)_2_][NTf_2_]_2_ and the studied ionic liquid [C_4_(MIm)_2_][NTf_2_]_2_, respectively.

The depression ∆*T*°_fus_ = *T*°_0_ − *T*°_1_ in the melting temperature of ionic liquid [C_4_(MIm)_2_][NTf_2_]_2_ indicates that the compound contains impurities. The total mole fraction of impurities (*x*_2_) in the studied sample was calculated by Equation (5):

−ln(1 − *x*_2_) = *A* · ∆*T*°_fus_ (1 + *B*·∆*T*°_fus_) (5)

where *A =* Δ_fus_*H*°/*R*(*T*°_fus_)^2^ and *B* = (*T*°_fus_)^−1^ − ½Δ*C*°_p_(*T*°_fus_)/Δ_fus_*H*° are the first and the second cryoscopic constants, respectively; Δ*C*°_p_(*T*°_fus_) is the heat capacity increase at the fusion of [C_4_(MIm)_2_][NTf_2_]_2_. The mole fraction of impurities in the studied DIL is equal to 0.0097.

The entropy of fusion Δ_fus_*S*° = (156.2 ± 1.7) J K^−1^ mol^−1^ of [C_4_(MIm)_2_][NTf_2_]_2_ was calculated using Equation (6):

Δ_fus_*S*° = _Δfus*H*_°/*T*°_fus_ (6)

Thus, the standard thermodynamic characteristics of fusion of [C_4_(MIm)_2_][NTf_2_]_2_ are *T*°_fus_ = (337.88 ± 0.01) K, Δ_fus_*H*° = (52.79 ± 0.28) kJ mol^−1^, and Δ_fus_*S*° = (156.2 ± 1.7) J K^−1^ mol^−1^, where the corresponding uncertainties are standard deviations of the mean.

The results of the present work were compared with the literature data [19,20,21,22,23]. It was confirmed that the *T*_fus_ values of the dicationic ILs are substantially higher than those of the monocationic ILs with the same anion [NTf_2_]. Thus, the dicationic ILs seem more suitable for applications at high-temperature conditions than the corresponding monocationic ILs. Earlier, the temperature and the enthalpy of fusion of geminal dicationic ionic liquid [C_4_(MIm)_2_][NTf_2_]_2_ were determined by the authors of [22] using differential scanning calorimetry (DSC). The melting point measured by DSC is reported as 329.2 K [22]. This value belongs to the temperature interval of the revealed phase transition. In addition, the value of Tm for dicationic ionic liquid [C_4_(MIm)_2_][NTf_2_]_2_ is equal to (332.5 ± 0.7) K in accordance with the data from [19]. The authors of [19] also performed DSC measurements with a heating rate of 10 K min^−1^ to determine the melting temperature of the above IL. In our work, the value of *T*°_fus_ = (337.88 ± 0.01) K was determined as the end temperature in the range of fusion by precise adiabatic calorimetry under equilibrium conditions. Therefore, in comparing the literature values [19,22] with those resulting from our study, a close agreement could not be expected. Moreover, we note that no details of DSC experiments and the evaluation of *T*_fus_ are provided in [19,22]. With regard to the enthalpy of fusion, admittedly, a rather large discrepancy is found between our value, (52.79 ± 0.28) kJ mol^−1^, and the value reported in [22] (23.6 kJ mol^−1^). The reason for this disagreement is difficult to understand. However, we note that the value of Δ_fus_*H* [22] seems anomalously low when compared with that of the monocationic counterpart (EMImNTf_2_), which was reported as (21.89 ± 0.03) kJ mol^−1^. For the sake of comparison, it can be noted that the Δ_fus_*H* values for the corresponding bromide compounds are 43.98 kJ mol^−1^ [22] and (18.26 ± 0.12) kJ mol^−1^ [23], for the dicationic and monocationic compounds, respectively.

### 2.3. Standard Thermodynamic Functions

The standard thermodynamic functions of [C_4_(MIm)_2_][NTf_2_]_2_ listed in Table 4 were calculated from the *C*_p_ values in the temperature range of 0–350 K. In order to determine the standard molar thermodynamic functions of [C_4_(MIm)_2_][NTf_2_]_2_, the Debye law [24] was used to extrapolate the low-temperature heat capacity from 6.5 K to 0:

*C*_p_ = *nD*(*Θ_D_*/*T*) (7)

where *n* is the number of degrees of freedom, *D_n_* = $\frac{n}{z^{n}}\int_{0}^{z}\frac{x^{n}}{\exp\left(x\right)-1}dx$ represents the Debye function, and *Θ_D_* is the Debye characteristic temperature. In Equation (7), the parameter *n* = 6, and *Θ_D_* = 38.8 K is an adjustable coefficient. The experimental heat capacity values of the investigated DIL in the range of 6.5–10 K were described by Equation (7) using the above parameters with an error of 1.87%. The values of [*H*°(*T*) − *H*°(0)] and those of *S*°(*T*) were calculated in the temperature range 0–350 K by numerical integrations of the *C*_p_ = *f*(*T*) and *C*_p_ = *f*(ln*T*) values, respectively, while those of [*G*°(*T*) − *H*°(0)] were determined according to Equation (8):

[*G*°(*T*) − *H*°(0)] = [*H*°(*T*) − *H*°(0)] − *TS*°(*T*) (8)

The results previously reported that the heat capacity of DILs is scarce and limited to temperatures higher than room temperature. No previous investigation was reported from temperatures close to 0 K up. The most investigated series of DILs is that with the bromide ion, which was considered for thermal storage applications [22]. One determination [22] is also available for [C_4_(MIm)_2_][NTf_2_]_2_, which is, to the best of our knowledge, the only *C*_p_ value ever reported for a DIL with the NTf_2_ anion before the present work. A direct comparison with our result is uncertain because the only value in [22] (1288 J K^−1^ mol^−1^) is given with no specification of the temperature. Assuming this value referred to the melting temperature (329.3 K in [22]), it exceeds our result by more than 20%. The few data available to date allow for some insight into the dependence of the heat capacity on the nature of the anionic portion. According to our results, the heat capacity of [C_4_(MIm)_2_][NTf_2_]_2_ is much lower than that of other DILs with the [C_4_(MIm)_2_]^++^ cation and typical anions such as BF_4_^–^, Br^–^ and PF_6_^–^, thus confirming the strong dependence of heat capacities from the anion. The observed *C*_p_ trend, Br^−^ ≅ BF_4_^−^ > PF_6_^−^ > NTf_2_^−^, can be readily interpreted as due to the different capacity to form hydrogen bonds. For example, a large number of hydrogen bonds was found in the crystal structure of C_4_(MIm)_2_]Br_2_ [22], also accounting for high values of the melting temperature and fusion enthalpy of this compound. Interestingly, the above heat capacity order is reversed if the decomposition temperatures are considered. It is thus interesting to wonder how accurately the measured *C*_p_ values for the liquid phase of [C_4_(MIm)_2_][NTf_2_]_2_ could be predicted by a simple estimation procedure based on the *C*_p_ of the corresponding monocationic compounds. From the structure shown in Figure 1, it comes natural to tentatively estimate the *C*_p_ of [C_4_(MIm)_2_][NTf_2_]_2_ as twice that of [C_2_MIm][NTf_2_] [25] corrected by the different contributions of the CH_3_ and CH_2_ groups. By using this correction the group contribution values reported by Zábranský and Růžička [26], the estimated *C*_p_ value of liquid [C_4_(MIm)_2_][NTf_2_]_2_ at the melting point (*C*_p_(est) = 992.0 J K^−1^ mol^−1^ at *T*_fus_) agrees fairly well (2.0%) with the experimental values reported in Table 2 (*C*_p_(exp) = 1012 J K^−1^ mol^−1^). If confirmed by further measurements, this simple procedure would allow obtaining a potentially useful, albeit rough, estimate of *C*_p_ for DILs for which no experimental value is available.

## 3. Materials and Methods

The structural formula of 1,4-bis(3-methylimidazolium-1-yl)butane bis(trifluoromethylsulfonyl)imide ([C_4_(MIm)_2_][NTf_2_]_2_) is presented in Figure 1 with *n* = 4. The synthesis and structural characterization of the studied dicationic ionic compound and of its bromide precursor has been carried out according to the procedure reported below (Section 3.1 and Section 3.2). The benchmark compound [C_4_MIm][NTf_2_] and lithium bis(trifluoromethylsulfonyl)imide Li[NTf_2_] were purchased by Sigma-Aldrich (purity > 99.0% is provided by the supplier) and used as such. The information for the studied dicationic ionic liquid [C_4_(MIm)_2_][NTf_2_]_2_ and the used reagents are listed in Table 5. The standard atomic masses recommended by the IUPAC Commission [27] were used to calculate the molar mass of the tested compound (*M*(C_16_H_20_F_12_N_6_O_8_S_4_) = 780.56 g mol^−1^).

The water content in the synthesized dicationic ionic liquid [C_4_(MIm)_2_][NTf_2_]_2_ and in the validation compound [C_4_MIm][NTf_2_] was checked by thermogravimetry (simultaneous Stanton Redcroft STA1500 at 10 °C/min under inert gas atmosphere), and it resulted in being below the detection limit of the instrument for both compounds (Figure S4, Supplementary Material). The mole fraction purity of the studied DIL was determined by the fractional melting technique in an adiabatic calorimeter to be 0.9903. Thus, it can be claimed that the purity of the synthesized DIL is high and meets the requirements of testing its thermodynamic properties.

### 3.1. Synthesis of the Bromide Precursor ([C_4_(MIm)_2_][Br]_2_)

1,4-Dibromobutane (10.15 g, 47 mmol) was slowly added into a round-bottom flask (100 mL) containing a stirred solution of 1-methylimidazole (7.725 g, 94 mmol) in ethyl acetate (50 mL). The reaction mixture was stirred at room temperature under an inert atmosphere (Ar) for 5 days. The white solid thus obtained was filtered on a Gooch (porosity IV) and then washed several times with ethyl acetate to remove the unreacted starting materials. The solid was dried first in a rotary evaporator (70 °C) for 3 h and then allowed under vacuum overnight at 70 °C to give the precursor 1,4-bis(3-methylimidazolium-1-yl)butane dibromide, [C_4_(MIm)_2_][Br]_2_, as a white solid (14.99 g, 39 mmol, 83%).

^1^H NMR (DMSO-*d*_6_, 300 MHz): δ = 9.29 (s, 2H), 7.83 (s, 2H), 7.75 (s, 2H), 4.25 (br, 4H), 3.84 (s, 6H), 1.79 (br, 4H). The provided results of NMR analysis (Figure S5, Supplementary Material) are in agreement with the literature data [16].

### 3.2. Synthesis of the Test Compound ([C_4_(MIm)_2_][NTf_2_]_2_)

A solution of 1,4-bis(3-methylimidazolium-1-yl)butane dibromide (4.0 g, 10.5 mmol) and lithium bis(trifluoromethylsulfonyl)imide (6.1 g, 21.2 mmol) in water (50 mL) was stirred at room temperature for 24 h. The lower liquid layer was separated and washed with seven 100 mL portions of water. After drying in a rotary evaporator, 5.65 g (7.24 mmol, 69%) of 1,4-bis(3-methylimidazolium-1-yl)butane bis(trifluoromethylsulfonyl)imide ([C_4_(MIm)_2_][NTf_2_]_2_) was obtained as a white solid.

^1^H NMR (DMSO-*d*_6_, 300 MHz): δ = 9.06 (br s, 2H), 7.71 (m, 2H), 7.69 (m, 2H), 4.20 (m, 4H), 3.86 (s, 6H), 1.80 (m, 4H).

^13^C NMR (DMSO-*d*_6_, 75 MHz): δ = 137.0, 124.0, 122.6, 119.9 (q, CF3, J = 320 Hz), 48.4, 36.1, 26.4.

The provided results of the NMR analysis (Figure S6, Supplementary Material) are in agreement with the literature data [16].

### 3.3. Heat Capacity Experiments

The heat capacity of dicationic ionic liquid [C_4_(MIm)_2_][NTf_2_]_2_ has been measured over the range from *T* = (6 to 350) K using a precise automatic BCT-3 adiabatic calorimeter. The calorimeter was manufactured by “Termis” joint-stock company (Moscow region, Russia). Its design and the operation procedure were described in detail elsewhere [28]. A miniature rhodium-iron resistance thermometer was calibrated on the basis of ITS-90 [29] and used for the temperature measurements during the calorimetric experiments. More detailed information about the thermometer calibration is published elsewhere [30,31]. The sensitivity of the thermometric circuit was 0.001 K. Liquid helium and nitrogen were used as cooling agents.

The measurements were performed at *p*_298_(He) = (4 ± 1) kPa in the calorimetric cell with the studied substance (dry helium used as the thermal exchange gas). A high vacuum inside the container was kept by means of cryosorption provided with an efficient charcoal getter. The mass of the sample loaded in a thin-walled cylindrical titanium ampoule of the BCT-3 device was (0.56495 ± 0.00001) g. After assembling, the measuring system was cooled in the Dewar vessel with liquid nitrogen. When the measurements were performed below 80 K, liquid helium was used. The studied sample was first measured in the liquid nitrogen region, followed by measurements in the liquid helium with overlapping experimental temperature intervals.

The calorimeter reliability was verified by measuring the heat capacities of the reference samples, namely high-purity copper (mass fraction 0.99999) [32], synthetic corundum α-Al_2_O_3_ (mass fraction 0.99993) [33], chromatographically pure *n*-heptane (mole fraction 0.99997) [34], and benzoic acid (mole fraction 0.99997) [35]. The test of the calorimeter revealed the combined expanded relative uncertainty of the experimentally determined heat capacity *U*_c,r_(*C*_p_) = 0.02 in the range *T* = (6–15) K, *U*_c,r_(*C*_p_) = 0.005 for *T* = (15–40) K, *U*_c,r_(*C*_p_) = 0.002 in the interval *T* = (40–350) K. The temperature and enthalpy of the phase transformations were determined with the standard uncertainty *u*(*T*_tr_) = 0.01 K and the combined relative expanded uncertainty *U*_c,r_(Δ_tr_*H*) = 0.008 and *U*_c,r_(Δ_tr_S) = 0.011, respectively. The reported uncertainties correspond to the 0.95 confidence level (*k* ≈ 2).

Note that the time required for the calorimeter to reach the equilibrium temperature was determined experimentally. These values are 40 min in the phase transition region (the equilibrium period) and 15 min in all other temperature intervals. When the duration decreases, the equilibrium is failed in the system. The heat capacity of the sample varied from (20 to 40)% of the total heat capacity of the (calorimetric ampoule + substance) system over the range *T* = (6–350) K.

## 4. Conclusions

This study reports the original results of the calorimetric study on 1,4-bis(3-methylimidazolium-1-yl)butane bis(trifluoromethylsulfonyl)imide dicationic ionic compound ([C_4_(MIm)_2_][NTf_2_]_2_). In particular, the heat capacity of this substance was measured for the first time in the temperature range of 6–350 K by precise adiabatic calorimetry. The thermodynamic characteristics of fusion were determined by the fractional melting experiments and the calorimetric method of continuous energy input. The melting temperature of [C_4_(MIm)_2_][NTf_2_]_2_ was found to be (337.88 ± 0.01) K, a value almost 70 K higher than that of the corresponding monocationic IL with a methyl and a butyl substituent on the imidazolium ring ([C_4_MIm][NTf_2_]). The *C*_p_ value of [C_4_(MIm)_2_][NTf_2_]_2_, in the liquid phase and referred to as the melting temperature, is only slightly overestimated (~3%) by a simple empirical value based on doubling the *C*_p_ of the [C_2_MIm][NTf_2_] monocationic IL (considered as “one half” of [C_4_(MIm)_2_][NTf_2_]_2_). Finally, the molar heat capacity values were used to calculate the standard thermodynamic functions (enthalpy [*H*°(*T*) − *H*°(0)], entropy *S*°(*T*), the Gibbs energy [*G*°(*T*) − *H*°(0)]) of [C_4_(MIm)_2_][NTf_2_]_2_ over the interval of *T* = (0 to 350) K.

## Figures and Tables

**Table 1:** The experimental molar heat capacity (in J K^−1^ mol^−1^) of dicationic ionic liquid [C_4_(MIm)_2_][NTf_2_]_2_ (*M*(C_16_H_20_F_12_N_6_O_8_S_4_) = 780.56 g mol^−1^) ^1^.

*T*/K	*C*_p_	*T*/K	*C*_p_	*T*/K	*C*_p_
Series 1					
6.52	14.1	17.14	67.49	47.73	220.5
6.85	14.7	17.61	70.68	49.37	226.6
7.22	16.1	18.09	73.60	51.01	232.6
7.52	17.1	18.57	76.49	52.66	238.5
7.81	18.3	19.05	79.79	54.31	244.5
8.21	19.6	19.53	83.09	56.40	251.7
8.74	21.4	20.01	86.29	58.11	257.3
9.21	23.7	20.99	92.56	60.12	263.9
9.78	25.8	22.45	102.1	61.81	269.1
10.33	28.2	23.94	110.9	63.62	274.8
10.89	30.3	25.45	120.0	65.11	279.4
11.35	32.7	26.98	128.7	66.31	282.8
11.84	35.0	28.51	137.8	67.63	286.7
12.14	36.4	30.07	145.0	69.30	291.2
12.61	38.8	31.64	153.3	71.17	296.8
13.08	41.9	33.21	161.1	73.23	302.3
13.44	43.5	34.80	167.8	75.42	307.6
13.90	46.1	36.39	175.0	78.25	315.9
14.35	49.0	37.99	182.2	81.23	323.8
14.81	52.0	39.60	188.5	83.84	330.3
15.27	55.41	41.21	195.0	87.31	338.1
15.73	57.90	42.84	201.1	90.88	347.3
16.20	60.93	44.46	207.8	93.85	355.0
16.67	64.26	46.35	215.2	96.81	362.0
Series 2					
83.12	328.0	149.78	484.5	236.12	669.0
85.67	334.8	151.97	489.5	240.86	678.9
87.77	340.0	154.14	493.8	245.59	688.3
89.88	344.9	156.31	498.1	250.33	700.1
91.99	350.4	158.47	503.9	255.07	711.4
94.10	355.4	160.64	509.6	260.91	725.5
96.21	360.6	162.82	513.8	265.13	736.3
98.33	365.9	164.99	517.5	269.88	747.5
100.45	371.2	167.17	522.9	274.63	760.7
102.57	376.5	169.34	527.7	279.39	773.3
104.69	380.8	171.52	532.1	284.15	787.4
106.81	386.3	173.70	537.0	288.89	804.2
108.94	390.9	175.88	541.1	293.65	817.5
111.07	395.9	178.06	545.3	298.38	834.1
113.20	401.3	180.24	550.0	303.10	850.9
115.34	405.8	182.42	554.7	307.81	870.5
117.49	411.0	184.61	560.0	312.50	890.8
119.63	415.6	186.82	566.0	317.18	(917.5)
121.77	421.4	189.01	569.8	321.84	(958.9)
123.91	425.8	191.20	574.4	326.35	(1190)
126.06	430.4	193.39	579.2	329.23	(3998)
128.21	435.7	196.12	586.2	331.24	(9457)
130.36	441.0	199.21	592.6	333.21	(12,079)
132.51	446.0	202.88	598.2	334.03	(15,155)
134.66	450.6	206.10	603.3	334.15	(23,042)
136.82	455.5	209.67	610.2	334.37	(9651)
138.98	460.0	213.71	619.0	336.21	1011
141.14	465.4	217.81	628.1	339.24	1013
143.29	469.6	222.02	637.8	342.21	1017
145.45	475.1	226.73	651.0		
147.62	480.1	231.41	659.7		
Series 3					
316.69	994	330.21	1007	345.41	1021
318.74	997	332.90	1008	347.51	1025
321.12	1000	335.42	1010	350.12	1032
323.49	1000	338.21	1013		
327.52	1006	342.42	1017		
Series 4					
266.13	738.0	327.28	1060	337.42	70,215
269.33	749.0	330.73	1280	337.48	72,541
271.92	754.0	331.80	1351	337.58	85,145
275.02	763.0	332.80	1460	337.58	120,451
278.42	771.0	334.65	1740	337.59	200,471
281.52	780.0	335.78	2011	337.60	243,321
284.34	788.0	336.52	4655	337.61	281,541
287.91	800.2	336.72	8858	337.64	180,451
290.31	808.0	336.82	9501	337.67	92,514
294.12	820.0	336.99	14,214	337.74	10,012
297.01	836.0	337.13	15,111	337.78	1010
308.54	880.0	337.24	22,154	338.82	1014
311.90	888.9	337.29	27,581	340.52	1015
316.27	915.9	337.37	30,145		
320.16	956.7	337.40	35,214		

^1^ The standard uncertainty for temperature *u*(*T*) = 0.01 K in the interval of *T* = (6.52 to 350.12) K in Series 1–4. The combined expanded relative uncertainty for the heat capacity *U*_c,r_(*C*_p_) = 0.02, 0.005, and 0.002 in the intervals of *T* = (6.52 to 15.27) K, *T* = (15.73 to 41.21) K, *T* = (42.84 to 350.12) K in Series 1–4, respectively. The reported uncertainties correspond to the 0.95 confidence level (*k* ≈ 2). Series 1: the *C*_p_ values correspond to the crystalline state. Series 2: the *C*_p_ values correspond to the crystalline state (*T* = (83.12–307.81) K) and the liquid state (*T* = (336.21–342.21) K); the *C*_p_ values in brackets correspond to an apparent heat capacity in the interval of fusion. Series 3: the *C*_p_ values correspond to the supercooled state (*T* = (316.69–335.42) K) and the liquid state (*T* = (338.21–350.12) K. Series 4: fractional melting experiment.

**Table 2:** The experimental determination of the enthalpy of fusion of the dicationic ionic liquid [C_4_(MIm)_2_][NTf_2_]_2_ (*M*(C_16_H_20_F_12_N_6_O_8_S_4_) = 780.56 g mol^−1^; *p*° = 0.1 MPa) ^1^.

*T*°_i_^1^/K	*T*°_f_^1^/K	Δ*H*°_1_/J	Δ*H*°_2_/J	Δ*H*°_3_/J g^−1^	Δ*H*°_4_/J g^−1^	Δ_fus_*H*°/J mol^−1^
281.21	342.58	155.52	78.35	63.21	6.108	52,520
280.34	345.24	162.98	82.91	64.08	9.466	53,220
285.32	347.15	157.51	79.19	59.08	12.09	52,655
					Mean value:	
					(52.79 ± 0.28) kJ mol^−1^	

^1^ Δ*H*°_1_ is the energy introduced at the heating of the calorimeter with the sample from the onset temperature (*T*°*_i_*) to the end temperature (*T*°*_f_*); Δ*H*°_2_ = $\int_{T{{}^{\circ}}_{i}}^{T{{}^{\circ}}_{f}}C_{\mathrm{cal}}dT$ is the energy supplied to heat the empty calorimeter (*C*_cal_ is the heat capacity of the calorimeter); Δ*H*°_3_ = $\int_{T{{}^{\circ}}_{i}}^{T{{}^{\circ}}_{fus}}{C{}^{\circ}}_{p}\left(\mathrm{cr}\right)dT$ and Δ*H*°_4_ = $\int_{T{{}^{\circ}}_{fus}}^{T{{}^{\circ}}_{f}}{C{}^{\circ}}_{p}\left(\mathrm{liq}\right)dT$ Are the energies supplied to heat the studied sample in crystalline and liquid states, respectively (*C*°_p_(cr) and *C*°_p_(liq)) the heat capacities of crystalline and liquid states of ionic liquid, respectively)? The sample mass was (0.56495 ± 0.00001) g. The enthalpy of fusion Δ_fus_*H*° was calculated by the following equation: Δ_fus_*H*° = [(Δ*H*°_1_ − Δ*H*°_2_)/*m* − Δ*H*°_3_ − Δ*H*°_4_] *M*.

**Table 3:** Thermodynamic data on the equilibrium fusion temperature (*T°F*) vs. the inverse fraction (*F*^−1^) of the melted [C_4_(MIm)_2_][NTf_2_]_2_.

*T*°*_F_* (exp.)/K	*F*	*F* ^−1^	*T*°*_F_* (calc.)/K
336.52	0.1276	7.835	336.52
336.72	0.1494	6.696	336.72
336.82	0.1645	6.078	336.82
336.99	0.2004	4.989	337.01
337.13	0.2350	4.255	337.14
337.24	0.2728	3.666	337.24
337.29	0.2943	3.398	337.29
337.37	0.3393	2.947	337.37
337.40	0.3538	2.826	337.39
337.42	0.3768	2.654	337.42
337.48	0.4528	2.209	337.50
337.58	0.5966	1.676	337.59
	1	1.000	337.70
	∞	0.000	337.88

**Table 4:** The standard thermodynamic functions of the dicationic ionic liquid [C_4_(MIm)_2_][NTf_2_]_2_ (*M*(C_16_H_20_F_12_N_6_O_8_S_4_) = 780.56 g mol^−1^) ^1^.

*T*/K	*C*°_p_(*T*)/J K^−1^ mol^−1^	[*H*°(*T*) − *H*°(0)]/kJ mol^−1^	*S*°(*T*)/J K^−1^ mol^−1^	−[*G*°(*T*) − *H*°(0)]/kJ mol^−1^
Solid				
5	7.46	0.00995	2.68	0.00342
15	53.2	0.290	29.20	0.148
20	86.12	0.6383	48.99	0.3415
25	117.5	1.149	71.64	0.6424
30	145.1	1.807	95.56	1.060
35	168.9	2.593	119.8	1.598
40	190.1	3.491	143.7	2.257
45	210.0	4.492	167.3	3.035
50	228.9	5.590	190.4	3.929
60	263.6	8.056	235.3	6.059
70	293.2	10.84	278.2	8.628
80	320.2	13.91	319.1	11.62
90	345.4	17.24	358.3	15.00
100	370.0	20.82	396.0	18.78
110	393.6	24.64	432.3	22.92
120	416.7	28.69	467.6	27.42
130	439.9	32.97	501.8	32.27
140	462.8	37.49	535.3	37.45
150	485.1	42.23	568.0	42.97
160	507.0	47.19	600.0	48.82
170	528.7	52.37	631.4	54.97
180	550.3	57.76	662.2	61.44
190	572.0	63.37	692.5	68.21
200	593.3	69.20	722.4	75.29
210	611.6	75.22	751.8	82.66
220	633.7	81.45	780.8	90.32
230	655.9	87.89	809.4	98.27
240	677.6	94.56	837.8	106.5
250	699.6	101.4	865.9	115.0
260	722.7	108.6	893.8	123.8
270	748.0	115.9	921.5	132.9
280	775.8	123.6	949.2	142.3
290	806.3	131.5	977.0	151.9
298.15	836.0	138.1	999.7	159.9
300	841.8	139.7	1005	161.8
310	874.4	148.3	1033	172.0
320	907.0	157.2	1061	182.5
330	939.6	166.4	1090	193.2
337.88	963.8	173.9	1112	201.9
Liquid				
337.88	1012	226.7	1269	201.9
340	1014	228.9	1275	204.6
350	1032	239.1	1304	217.5

^1^ The expanded uncertainty for pressure *U*(*p*°) = 0.5 kPa. The standard uncertainty for temperature *u*(*T*) = 0.01 K between *T* = 6 K and *T* = 350 K. The combined expanded relative uncertainties *U*_c,r_(*C*°_p_ (*T*)) = 0.02, 0.005, and 0.002; *U*_c,r_([*H*°(*T*) – *H*°(0)]) = 0.022, 0.007, and 0.005; *U*_c,r_([*S*°(*T*) – *S*°(0)]) = 0.023, 0.008, and 0.006; *U*_c,r_([*G*°(*T*) − *H*°(0)]) = 0.03, 0.01, and 0.009 in intervals of *T* = (6 to 15) K, *T* = (15 to 40) K, and *T* = (40 to 350) K, respectively. The reported uncertainties correspond to the 0.95 confidence level (*k* ≈ 2).

**Table 5:** Sample information.

Chemical Name	Source	Purification Method	Purity/%	Analysis Method
1,4-bis(3-methylimidazolium-1-yl)butane bis(trifluoromethylsulfonyl)imide [C_4_(MIm)_2_][NTf_2_]_2_	Synthesis	Repeated washing with water; drying in vacuum	>99.0 (mole fraction)	^1^H NMR spectroscopy, TG analysis, adiabatic calorimetry
1,4-bis(3-methylimidazolium-1-yl)butane dibromide [C_4_(MIm)_2_][Br]_2_		Repeated washing with ethyl acetate; drying in vacuum		^1^H NMR spectroscopy
1-butyl-3-methylimidazolium bis(trifluoromethylsulfonyl)imide [C_4_MIm][NTf_2_] CAS No.: 174899-83-3	Sigma-Aldrich	Without any purification	>99.0 (mass fraction)	—
lithium bis(trifluoromethylsulfonyl)imide Li[NTf_2_] CAS No.: 90076-65-6				
1,4-dibromobutane C_4_H_8_Br_2_ CAS No.: 110-52-1				
1-methylimidazole C_4_H_6_N_2_ CAS No.: 616-47-7		Purified by distillation		

## Data Availability

Data are contained within this article or Supplementary Materials.

## Supplementary Materials

The following supporting information can be downloaded at: https://www.mdpi.com/article/10.3390/molecules29174180/s1, Figure S1: The molar heat capacity of the prototypic 1-butyl-3-methylimidazolium bis(trifluoromethyl)sulfonylimide [C_4_MIm][NTf_2_] (monocationic ionic liquid); Figure S2: Percentages of deviation of Ref. [15] data of the heat capacity (*C*_p_,_lit_) for [C_4_MIm][NTf_2_] from the values of the present work (*C*_p_); Figure S3: Percentages of deviation of the experimental heat capacity of [C_4_(MIm)_2_][NTf_2_]_2_ from the fitting values; Figure S4: TG curves of ionic liquids [C_4_MIm][NTf_2_] and [C_4_(MIm)_2_][NTf_2_]_2_; Figure S5: ^1^H NMR spectrum of [C_4_(MIm)_2_][Br]_2_ (DMSO-*d*_6_, 300 MHz); Figure S6: (A) ^1^H NMR spectrum of [C_4_(MIm)_2_][NTf_2_]_2_ (DMSO-*d*_6_, 300 MHz), (B) ^13^C NMR spectrum of [C_4_(MIm)_2_][NTf_2_]_2_ (DMSO-*d*_6_, 75 MHz); Table S1: Experimental molar heat capacity (in J K^−1^ mol^−1^) of [C_4_MIm][NTf_2_]; Table S2: Polynomial-fitting coefficients of the temperature dependence of the molar heat capacity of [C_4_(MIm)_2_][NTf_2_]_2_.
