# Supplementary material for: Thermodynamic Study of 1,4-Bis(3-methylimidazolium-1-yl)butane Bis(trifluoromethylsulfonyl)imide ([C4(MIm)2][NTf2]2) from 6 to 350 K
Source: Molecules. 2024 Sep 3;29(17):4180. doi: 10.3390/molecules29174180 (PMC11396955; doi:10.3390/molecules29174180)
Supplement: Supplementary file 1 [file molecules-29-04180-s001.zip › molecules-3161126-supplementary.pdf]

Thermodynamic Study of 1,4-Bis(3-methylimidazolium-1-yl)butane Bis(trifluoromethylsulfonyl)imide ([C4(MIm)2][NTf2]2) from 6 to 350 K

Alexey V. Markin, Andrea Ciccioli, Andrea Lapi, Semen S. Sologubov, Natalia N. Smirnova and Stefano Vecchio Ciprioti

Supplementary Materials

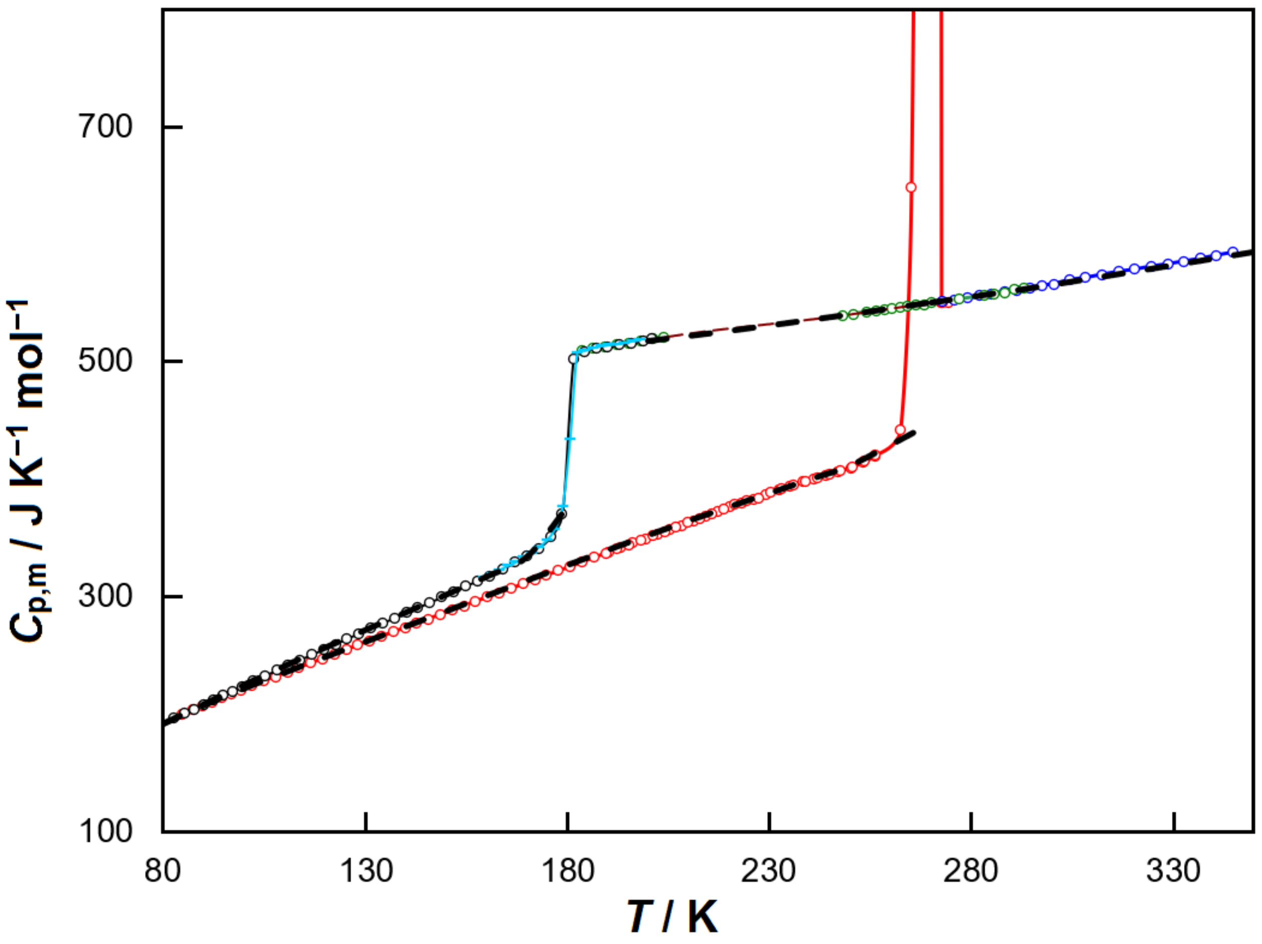

Figure S1. The molar heat capacity of the prototypic 1-butyl-3-methylimidazolium bis(trifluoromethyl)sulfonylimide [C_4MIm][NTf_2] (monocationic ionic liquid). The bold dashed lines (———) correspond to results of Ref. [15].

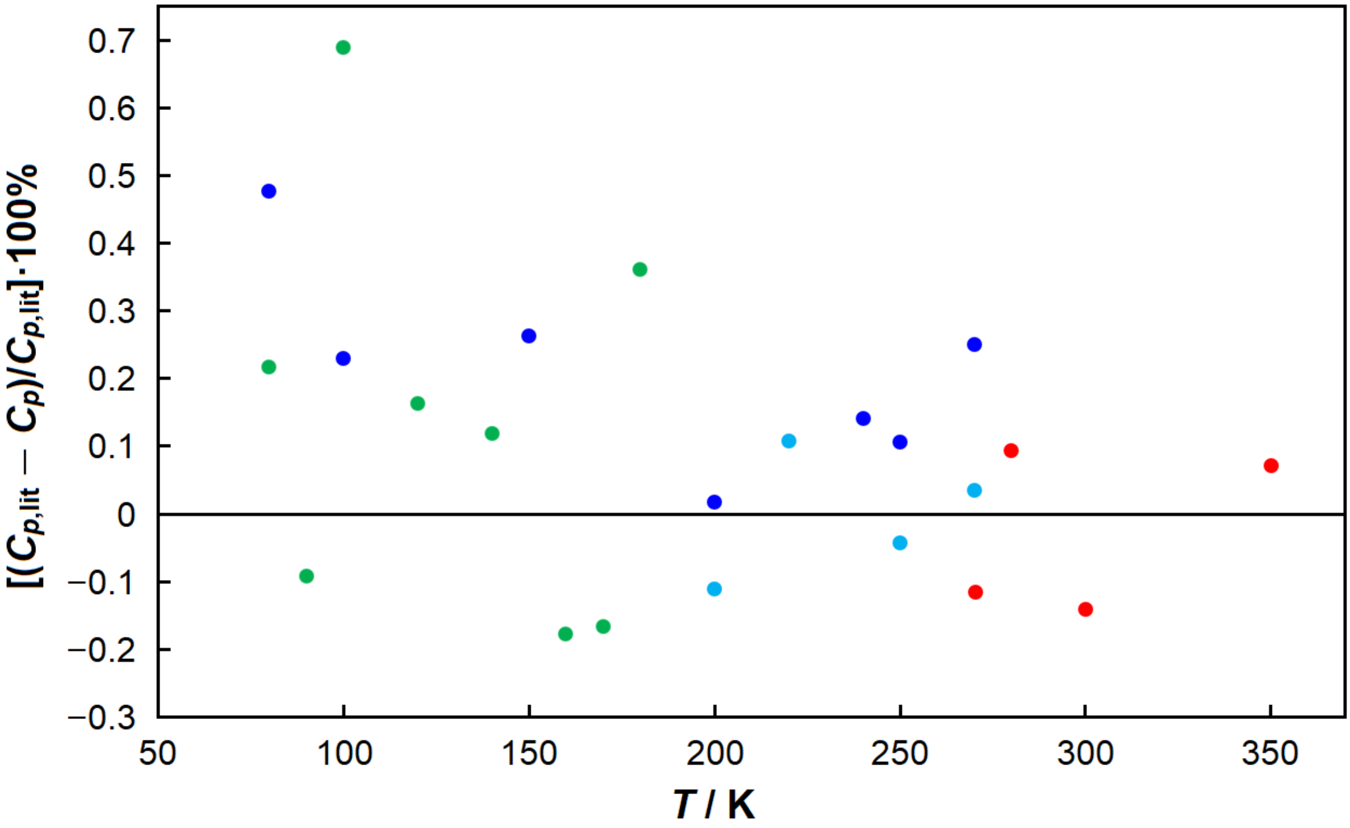

Figure S2. Percentages of deviation of Ref. [15] data of the heat capacity ($C_{p,\mathrm{lit}}$) for $[C_4MIm][NTf_2]$ from the values of the present work (C_p):
● the crystalline state; ● the liquid state; ● the glassy state; ● the supercooled liquid state.

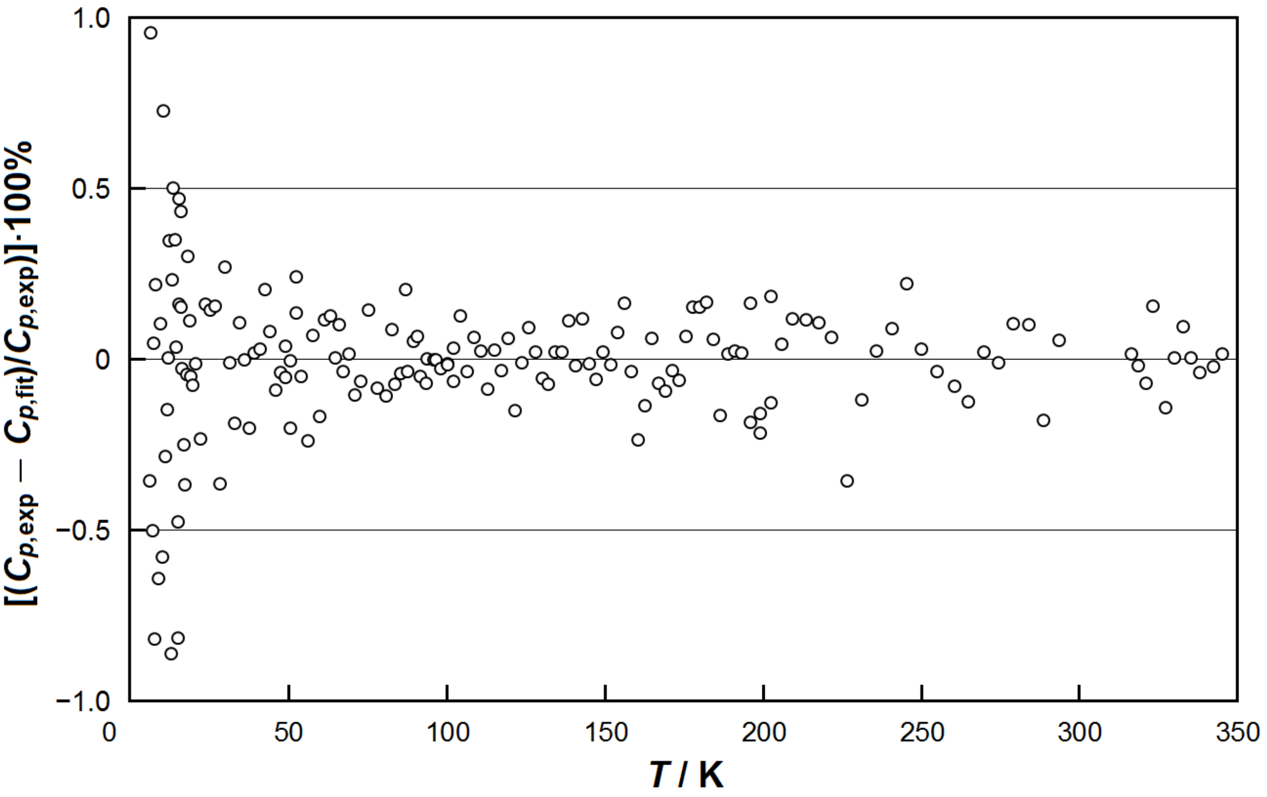

Figure S3. Percentages of deviation of the experimental heat capacity of $[C_4(MIm)_2][NTf_2]_2$ from the fitting values.

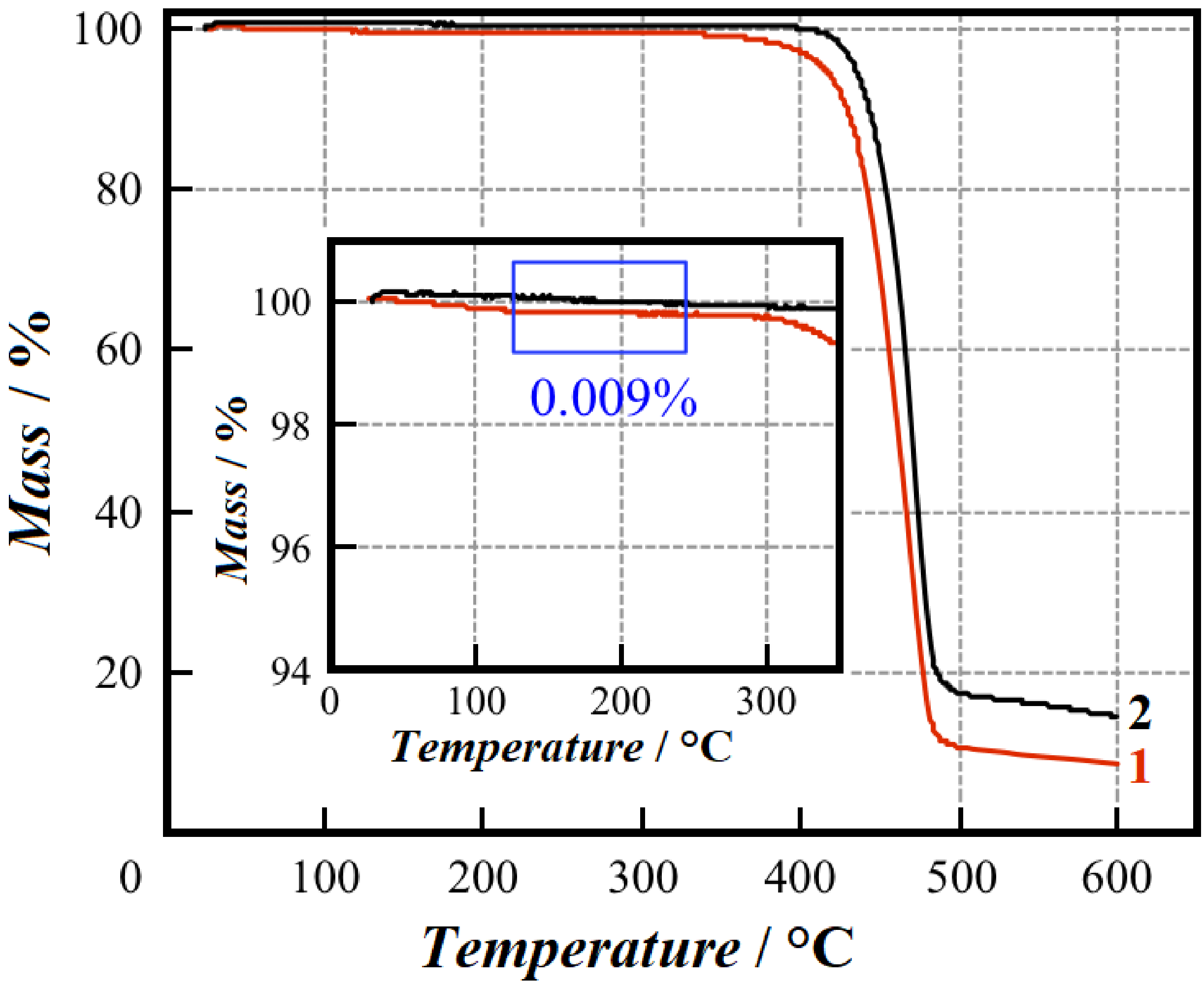

Figure S4. TG curves of ionic liquids [C_4MIm][NTf_2] (**1**) and [$C_4(MIm)_2$][NTf_2]$_2$ (**2**).

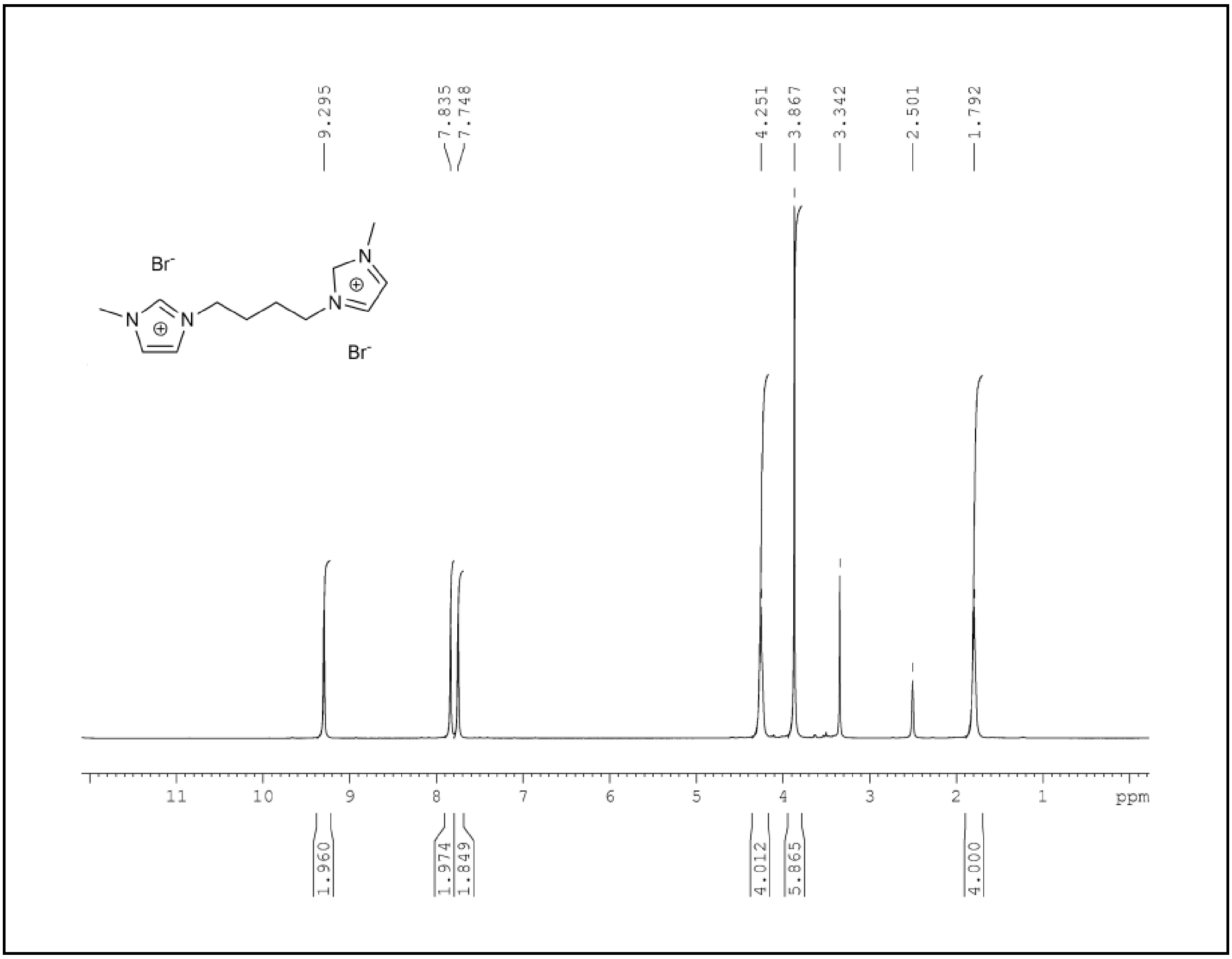

Figure S5. ^{1}H NMR spectrum of $[C_4(MIm)_2][Br]_2$ (DMSO-d_6, 300 MHz): the peak at 3.3 ppm is attributed to water (always present in DMSO); the peak at 2.5 ppm is due to DMSO solvent.

(A)

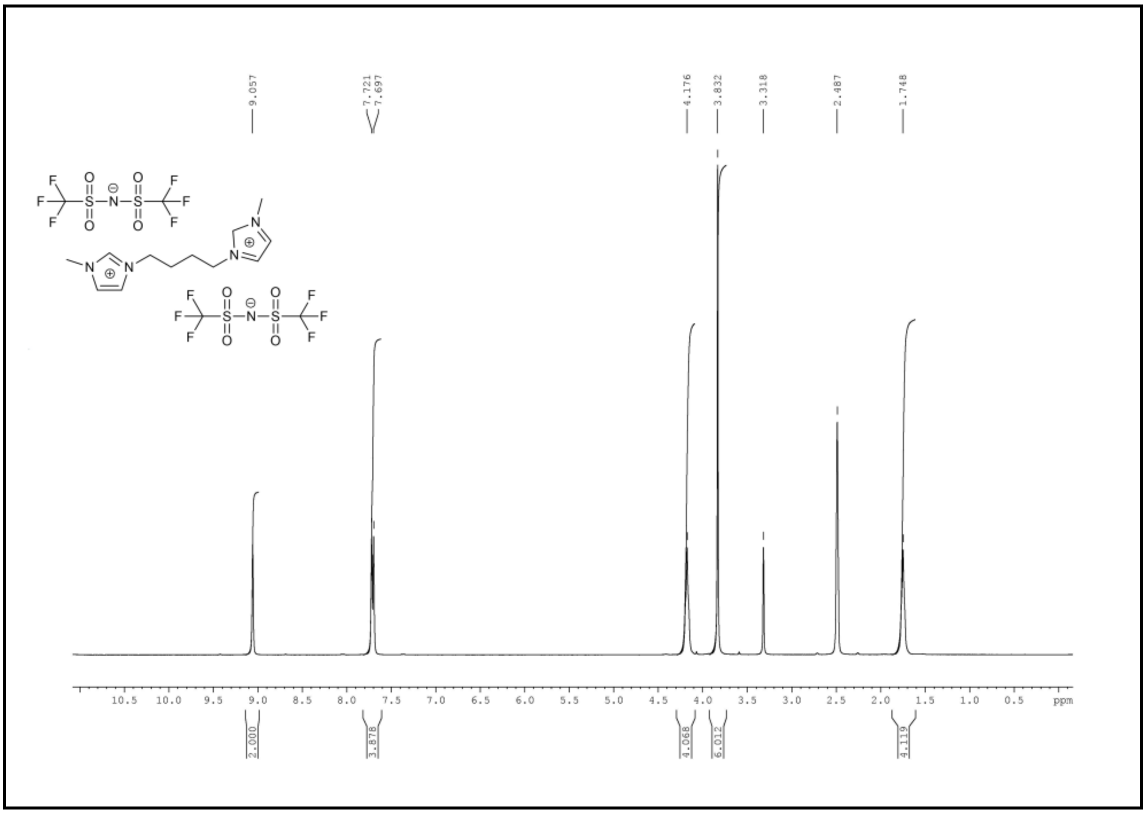

(B)

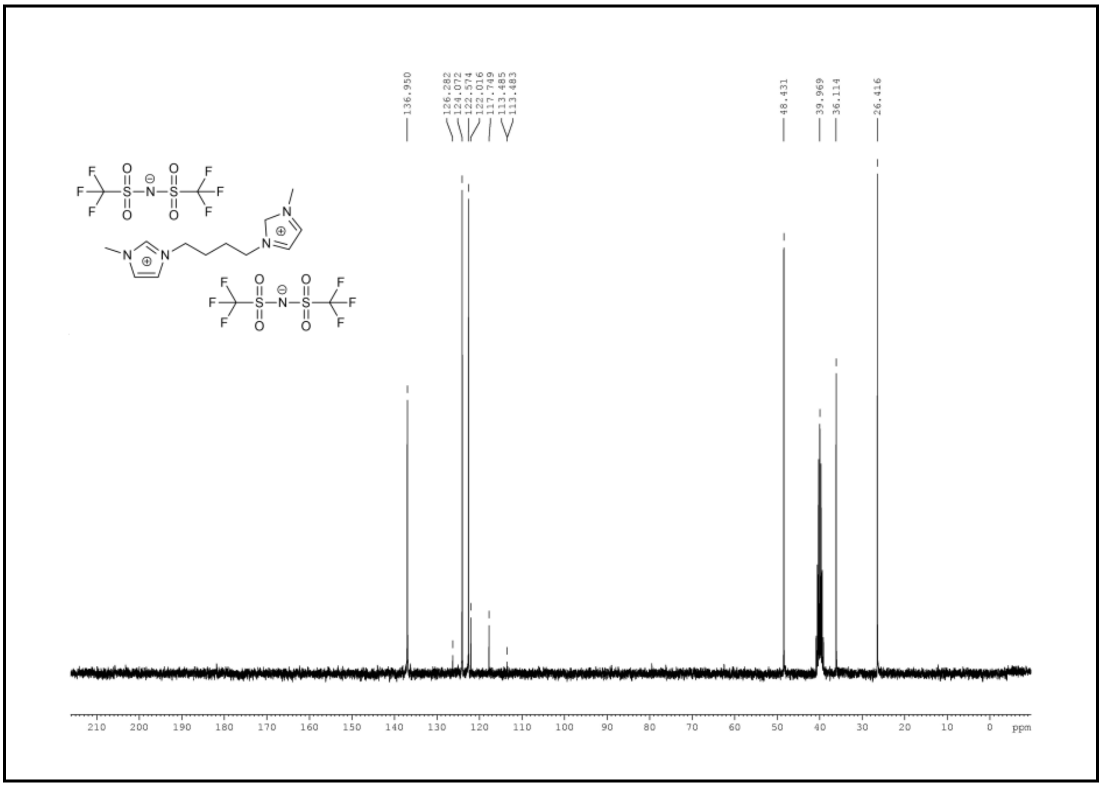

Figure S6. (A) ^{1}H NMR spectrum of $[C_4(MIm)_2][NTf_2]_2$ (DMSO-d_6, 300 MHz): the peak at 3.3 ppm is attributed to water (always present in DMSO); the peak at 2.5 ppm is due to DMSO solvent; **(B)** ^{13}C NMR spectrum of $[C_4(MIm)_2][NTf_2]_2$ (DMSO-d_6, 75 MHz).

Table S1. Experimental molar heat capacity (in J K^{-1} mol^{-1}) of $[C_4MIm][NTf_2]^{a,b}$.

T/K	$C_{p,m}$	T/K	$C_{p,m}$	T/K	$C_{p,m}$
**Series 1**		195.90	516.6	119.46	247.8
82.65	197.0	198.94	518.8	122.37	251.6
85.18	201.4	200.94	520.3	125.29	255.4
87.58	205.0	**Series 2**		128.21	259.4
89.98	209.0	190.10	338.4	131.12	263.1
92.39	212.8	193.27	342.3	134.04	266.8
94.79	216.5	196.25	346.1	136.95	270.6
97.20	220.2	199.33	349.9	139.87	274.2
99.61	224.0	202.49	354.0	142.79	278.0
102.26	228.8	205.45	357.8	145.71	281.7
105.15	233.3	208.41	361.3	148.62	285.5
108.05	237.9	211.37	365.3	151.54	289.2
110.95	242.3	214.34	369.4	154.46	292.8
113.86	246.8	217.30	373.1	157.38	296.7
116.77	251.3	220.28	377.1	160.29	300.3
119.68	255.7	223.25	380.7	163.21	303.9
122.60	260.2	226.22	383.8	166.13	307.6
125.53	265.0	229.20	387.4	169.05	311.4
128.45	269.3	232.54	391.4	171.97	315.0
131.37	273.8	235.15	394.6	174.90	319.0
134.29	278.1	238.32	398.3	177.82	322.6
137.21	282.6	241.16	401.1	180.74	326.2
140.14	287.0	244.15	404.0	183.66	329.9
143.07	291.4	246.94	407.0	186.58	333.9
146.00	295.8	250.21	410.3	189.49	337.5
148.93	300.2	253.20	415.0	192.41	341.1
151.86	304.9	256.19	420.5	195.33	344.7
154.80	309.6	**Series 3**		198.24	348.4
157.73	314.2	84.81	200.4	201.16	352.5
160.86	318.5	87.36	204.1	204.07	356.0
164.05	323.9	89.76	207.7	206.99	359.5
166.97	329.8	92.16	211.2	209.90	363.5
169.92	335.5	94.57	214.5	212.81	367.2
172.87	342.0	96.98	217.7	215.72	371.2
(175.83)	(351.5)	99.39	220.7	218.63	375.1
(178.76)	(371.3)	102.04	224.8	221.55	378.9
(181.50)	(502.9)	104.94	228.7	224.46	382.3
184.20	509.5	107.84	232.6	227.37	384.7
187.11	512.3	110.74	236.4	230.26	389.3
190.07	513.4	113.65	240.3	233.17	392.2
193.02	515.2	116.55	244.1	236.08	395.9

T/K	$C_{p,m}$	T/K	$C_{p,m}$	T/K	$C_{p,m}$
239.00	399.0	304.32	570.1	198.07	519.0
241.90	402.0	308.38	572.1	**Series 6**	
244.80	405.0	312.44	574.7	183.61	509.6
247.71	408.0	316.49	577.3	186.47	511.8
248.99	410.3	320.54	579.1	189.42	513.6
250.61	411.0	324.59	581.7	192.36	515.0
251.45	414.3	328.64	583.6	195.30	516.6
253.50	415.7	332.69	586.0	198.24	518.1
253.97	417.2	336.73	588.4	201.18	520.0
256.38	420.8	340.78	591.2	203.88	521.4
(256.81)	(421.3)	344.82	593.7	**Series 7**	
(258.52)	(427.1)	**Series 5**		277.08	553.7
(259.46)	(430.3)	159.22	316.8	283.32	556.9
(262.47)	(442.2)	161.31	320.1	285.79	558.5
(265.19)	(648.8)	163.26	323.2	288.27	559.7
(267.39)	(1848)	165.21	326.8	290.74	561.7
(269.08)	(7366)	167.16	329.8	293.21	562.9
(269.80)	(17939)	169.12	333.8	**Series 8**	
(270.00)	(19235)	171.08	337.9	248.28	539.6
(270.20)	(551.2)	173.04	342.8	250.81	541.1
272.61	551.4	(175.00)	(348.7)	254.15	543.0
274.51	551.3	(176.96)	(358.2)	256.51	544.2
**Series 4**		(178.90)	(377.4)	257.40	544.6
272.90	551.5	(180.77)	(434.5)	258.50	545.1
275.92	552.6	(182.51)	(507.8)	260.44	546.2
279.01	555.2	184.40	510.0	262.42	546.9
282.09	556.9	186.36	512.4	264.38	547.8
285.18	558.5	188.31	513.8	266.36	548.5
288.27	560.2	190.27	514.8	268.34	549.3
294.45	563.6	192.22	515.6	270.32	550.4
297.55	565.3	194.17	516.7	272.65	551.2
300.64	566.8	196.12	518.1		

[a] The standard uncertainty for temperature $u(T)$ = 0.01 K in the interval of T = (82.65 to 344.82) K. The combined expanded relative uncertainty for heat capacity $U_{c,r}(C_{p,m})$ = 0.002 in the above temperature range. The reported uncertainty corresponds to the 0.95 confidence level ($k \approx 2$).

[b] **Series 1:** the $C_{p,m}$ values correspond to the glassy state (T = (82.65–172.87) K), the supercooled liquid state (T = (184.20–200.94) K; the $C_{p,m}$ values in brackets correspond to an apparent heat capacity.

Series 2: the $C_{p,m}$ values correspond to the crystalline state.

Series 3: the $C_{p,m}$ values correspond to the crystalline state (T = (84.81–256.38) K); the $C_{p,m}$ values in brackets correspond to an apparent heat capacity; the $C_{p,m}$ values correspond to the liquid state (T = (272.61–274.51) K).

Series 4, 7: the $C_{p,m}$ values correspond to the liquid state.

Series 5: the $C_{p,m}$ values correspond to the glassy state (T = (159.22–173.04) K), the supercooled liquid state (T = (184.40–196.12) K; the $C_{p,m}$ values in brackets correspond to an apparent heat capacity.

Series 6, 8: the $C_{p,m}$ values correspond to the supercooled liquid state.

Table S2. Polynomial-fitting coefficients of the temperature dependence of the molar heat capacity of $[C_4(MIm)_2][NTf_2]_2$.

ΔT/K	6–16	15–53	49–103	100–203	196–294
Polynomial equation	(1)	(2)	(2)	(2)	(3)
Polynomial coefficients A_i/J K^{-1} mol^{-1}					
A_1	4.760200	145.0760	−122.9198	−32983.37	119404.8
A_2	2.288576	153.4601	1469.117	131561.9	−83439.00
A_3	10.80568	7.779954	−2001.676	−215738.4	24211.51
A_4	28.12178	−10.81151	316.0705	187712.2	−3720.464
A_5	31.07333	65.45318	2064.665	−91281.34	319.5983
A_6	15.88593	41.83592	−1943.794	23533.65	−14.55646
A_7	3.096037	−9.449229	542.7701	−2512.879	0.2748003
